# Pineal region metastasis with intraventricular seeding: A case report and literature review

**DOI:** 10.1097/MD.0000000000016652

**Published:** 2019-08-23

**Authors:** Junpeng Ji, Chunyu Gu, Mingshan Zhang, Hongwei Zhang, Haoran Wang, Yanming Qu, Ming Ren, Weihai Ning, Chunjiang Yu

**Affiliations:** Department of Neurosurgery, Sanbo Brain Hospital, Capital Medical University, Beijing, China.

**Keywords:** intraventricular seeding, metastasis, pineal region

## Abstract

**Introduction:**

Tumors of the pineal region are rare, and metastatic carcinoma occurring in the pineal region is extremely rare. No previous reports have described pineal region metastasis with intraventricular seeding.

**Patient concerns:**

We report a case of a 51-year-old woman presented with a 1-week history of severe headache, nausea, and vomiting. Imaging examination revealed 2 lesions in the pineal region and the right lateral ventricle.

**Diagnosis:**

Pinealocytoma or germinoma was considered as the preoperative diagnosis. The postoperative pathological diagnosis was small cell neuroendocrine carcinoma. After bronchoscopic biopsy, small cell lung cancer was confirmed.

**Interventions:**

A right frontal craniotomy and a translateral ventricle approach were performed to remove 2 lesions completely. And regular radiotherapy and chemotherapy were initiated after surgery.

**Outcomes:**

The patient was discharged from the hospital 2 weeks after operation and went to another cancer hospital for bronchoscopic biopsy, radiotherapy, and chemotherapy. Finally, the patient died 2 years after surgical treatment.

**Conclusion:**

Metastatic tumors of the pineal region are very rare. For patients with pineal lesions, a diagnosis of a metastatic tumor should be considered. Retrograde cerebrospinal fluid circulation might be the reason for a secondary metastasis.

## 1 Introduction

Tumors of the pineal region are rare, accounting for less than 1% of primary intracranial lesions.^[1]^ Germ cell tumors, such as germinomas, teratomas, and yolk sac tumors, are the most frequent pineal tumors and others include pineal parenchymal tumors, glial tumors, cysts, meningiomas, and lipomas.^[2]^ However, metastatic neoplasms located in the pineal region are extremely uncommon. Lung cancer is the most common source of brain metastasis. Small cell lung cancer (SCLC) accounts for approximately 14% of all lung cancers,^[3]^ characterized by rapid progression and early metastasis.^[4]^ Brain metastases are most commonly found at the junction of the hemispheric gray and white matter and are overrepresented in “watershed” areas of the brain.^[5]^ To the best of our knowledge, no studies have reported pineal metastasis with intraventricular seeding. Here, we present a case of pineal region metastasis with a seeding lesion in the right lateral ventricle along with a review of 27 clinical cases^[6–30]^ of pineal metastasis. This research was approved by the Ethics Committee of Sanbo Brain Hospital, and informed written consent was obtained from the patient for publication of this case report and accompanying images.

## 2 Case presentation

**Figure 1:**
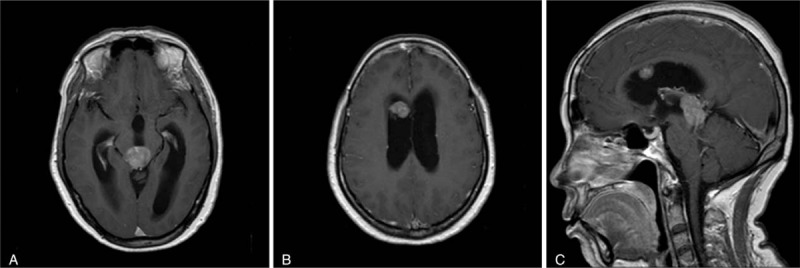
Preoperative contrast T1-weighted MRI showing enhancing lesions in the pineal region (A) and right lateral ventricle (B). Both lesions were visible via sagittal view (C). The lesion demonstrated a heterogeneous enhancement pattern.

A 51-year-old woman presented with a 1-week history of severe headache, nausea, and vomiting. Physical examination showed no convergence reflex and difficulty with upward gaze. Brain computed tomography (CT) scan revealed hydrocephalus and 1 lesion in the anterior horn of the right lateral ventricle and another in the posterior of the third ventricle. Magnetic resonance imaging (MRI) of the tumors demonstrated low signal intensity on T1-weighted images and high signal intensity on T2-weighted images. The lesions enhanced heterogeneously after the administration of contrast media. No obvious edema was seen in the imaging examination (Fig. 1). No obvious abnormalities were detected in blood or urine tests. Alpha fetoprotein (AFP) and human chorionic gonadotropin β (hCGβ) were negative. Hormone levels and chest X-ray were normal.

**Figure 2:**
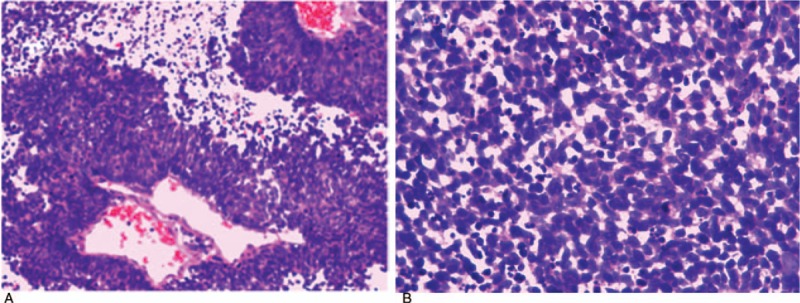
At 100× magnification (A), we observed tumor cells to be densely distributed in patches or around blood vessels, with a papillary arrangement. At 400× magnification (B), the structure of the tumor cells was clearly visible. Less cytoplasm, as well as hyperchromatic and oval nuclei, were observed in tumor cells, accompanied by apoptosis and thanatosis. Diagnosis: small cell malignant tumor.

Pinealocytoma or germinoma was considered as the preoperative diagnosis. A right frontal craniotomy and a translateral ventricle approach were performed to remove 2 lesions completely. A third ventriculostomy was performed to relieve hydrocephalus. The postoperative pathology diagnosis was pineal metastasis of small cell neuroendocrine carcinoma (Fig. 2). Immunohistochemical staining for thyroid transcription factor-1 (TTF-1) was positive, suggesting a primary site in the lung. Then, a chest CT scan confirmed a lesion in the left upper lung.

The patient was discharged from the hospital without serious complications after operation and went to another cancer hospital for bronchoscopic biopsy. SCLC was confirmed, and regular radiotherapy and chemotherapy were initiated. After 2 years of follow-up, the patient died due to deterioration of her general condition.

## 3 Discussion

Metastatic tumors that occur in the pineal region are extremely rare and are typically identified via autopsy.^[11]^ For example, Tsukada^[31]^ found 14 (4.5%) cases of pineal gland metastasis among 309 cases of central nervous system metastasis from breast carcinoma. However, in clinical practice, the proportion of patients suffering from pineal region metastasis is far below the above rate. Metastasis to the pineal region has been estimated^[32]^ to account for only 0.4% of all intracranial metastatic tumors from lung adenocarcinoma in Japan. We searched PubMed using the term “pineal region metastasis”, and 27 clinical cases in 25 English-language reports were reviewed. Among these cases, the lung was the primary site in 14 cases, 4 cases originated from esophagus, 2 cases from the stomach, 2 cases from the kidney, and the remaining 5 cases from the breast, colorectum, bladder, thyroid or liver. There were 7 female patients and 20 male patients. The average age was 59.22 ± 13.94 years. Nearly half of the patients had no extracranial malignant tumor history when the metastastic pineal region lesions were discovered. No reports of pineal region metastasis together with intraventricular seeding were found. The present report likely represents the first case of a pineal gland metastasis with intraventricular seeding.

The main clinical manifestation of pineal region metastasis was high intracranial pressure, which may lead to headache, nausea, vomiting, and vision loss. These symptoms were mainly caused by hydrocephalus. The clinical manifestations of the aforementioned patients included typical symptoms of increased intracranial pressure on admission, and Parinaud's syndrome was discovered during physical examination. Of the 27 clinical cases, 16 (59.26%) showed symptoms of increased intracranial pressure, 8 (29.62%) showed Parinaud's syndrome, 9 (33.33%) showed gait difficulty, and 2 (7.41%) showed no obvious symptoms (Table 1).

**Table 1:**
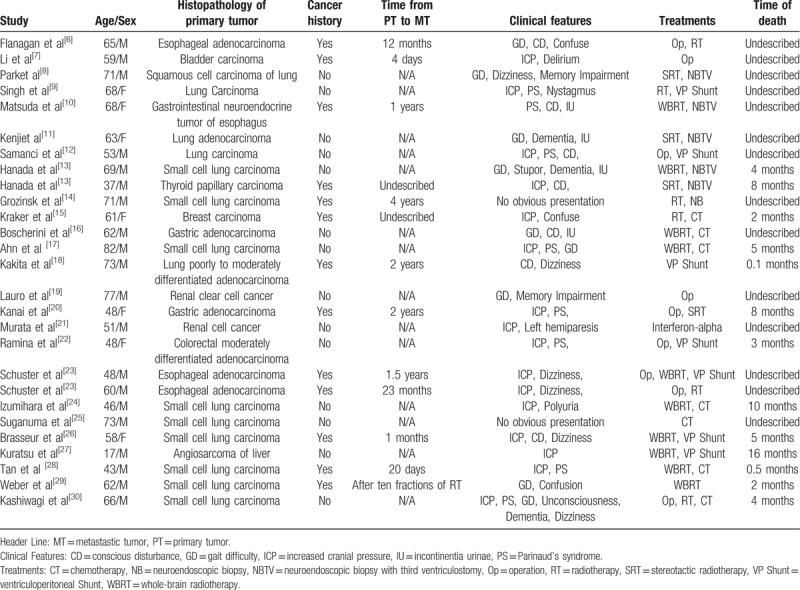
Overview of 27 cases of pineal metastases.

The pineal region is an extremely rare site of intracranial metastasis. The main route by which extracranial malignant tumors reach the pineal region is through hematogenous metastasis. The blood-brain barrier is weaker in the pineal region and circulating tumor cells are thus more likely to have access to the nervous system through this site to form metastases. Kashiwagi et al^[30]^ believed that the pineal region, which is full of numerous sinusoidal vessels without perivascular glial sheets, is more susceptible to circulating tumor cells in the blood. Ortega et al^[33]^ suggested that metastasis to the pineal region is mainly due to tumor cells entering the pineal region via the posterior choroidal artery. In our case, the lesion located in the pineal region was considered the primary metastasis, while the lesion located in the right ventricle was considered the secondary metastasis. The reason for the secondary metastasis might be turbulence in the cerebrospinal fluid during circulation. Pollack et al^[34]^ summarized the potential mechanism of tumor dissemination as follows: lesions arising in close proximity to the ventricles and basal cisterns show a predilection for CSF seeding. Operative manipulation and select biological factors, such as tumor consistency and intercellular adhesiveness, also play a role in CSF seeding.

Primary pineal region tumors, though rare, can originate from a variety of cell sources, such as pineal parenchymal tumors, germ cell tumors, astrocytomas, ependymomas, and papillary pineal tumors.^[35]^ Diagnosing metastasis in the pineal region is difficult without an explicit history of extracranial malignancies and often requires surgery or biopsy to reach a pathology diagnosis. Differential diagnosis through imaging, based on CT and MRI, is very difficult. Of the 27 clinical cases we reviewed, the metastastic lesions showed different degrees of enhancement, such as heterogeneous enhancement, peripheral enhancement, significant enhancement, or less obvious enhancement. The typical manifestation of brain metastasis is " small lesions with large edema”, potentially because the aggressive growth of the tumor destroys the blood-brain barrier and leads to vasogenic edema. In addition, the structure of the gray matter is compact in the brain, while the outer space of white matter is loose; therefore, edema of metastases mostly occurs in the white-matter area.^[36]^ However, the pineal region is lacking in gray matter, which may be why no obvious edema was observed on CT or MRI in our case.

Metastasis of the central nervous system is usually an indication of end-stage disease, and the treatment of pineal region metastases varies according to systemic conditions, pathology, and neurological symptoms. As a therapeutic strategy in this case, we performed tumor resection and third ventriculostomy. Afterwards, the patient received radiotherapy and chemotherapy. The 27 clinical cases we reviewed also received proper treatments based on patient state, such as radiotherapy (with or without biopsy) only or radiotherapy combined with chemotherapy (Table 1).

Of the 27 clinical cases, the outcomes was known for 13 cases. Of these, the survival period was 5.12 ± 4.43 (median 4) months. The reported^[37]^ survival period of brain metastasis is usually less than 24 months, and patients (KPS ≥ 70) with a single brain metastasis may achieve long-term survival with aggressive treatment. The survival period of the patient in our case was 21 months. Total resection of the tumor with radiotherapy and chemotherapy seemed to prolong the prognosis of the patient. A third ventriculostomy also helped to relieve hydrocephalus when the tumor relapsed, which may be another reason for her long survival. In a randomized trial investigating treatments of brain metastasis by Patchell et al,^[38]^ the overall survival of the surgery with radiation group was significantly longer than that of the single radiation group (40 weeks vs 15 weeks; *P* < .01). An optimal therapeutic effect can be achieved by combining surgery and radiotherapy.

## 4 Conclusion

The pineal gland is an extremely rare site for brain metastases. We presented the case of a 51-year-old woman with pineal region metastasis with intraventricular seeding. In addition, 27 cases of pineal region metastasis were reviewed. Metastasis is characterized by a lack of specificity, which makes it difficult to distinguish metastases from other tumors located in the pineal region through clinical and imaging approaches alone. Therefore, for patients with pineal region lesions, especially elderly patients, a diagnosis of a metastatic tumor should be considered. Retrograde cerebrospinal fluid circulation might be a reason for secondary metastasis. Larger studies are required to assess the molecular mechanisms.

## Author contributions

**Conceptualization:** Junpeng Ji.

**Data curation:** Junpeng Ji.

**Formal analysis:** Junpeng Ji.

**Funding acquisition:** Chunyu Gu.

**Investigation:** Junpeng Ji.

**Project administration:** Chunyu Gu, Chunjiang Yu.

**Resources:** Chunyu Gu.

**Supervision:** Chunyu Gu, Mingshan Zhang, Hongwei Zhang, Haoran Wang, Yanming Qu, Ming Ren, Weihai Ning, Chunjiang Yu.

**Validation:** Chunjiang Yu.

**Visualization:** Junpeng Ji.

**Writing – original draft:** Junpeng Ji.

**Writing – review & editing:** Chunyu Gu.
